# HIV and HCV among drug users and people living in prisons in Germany 2022: WHO elimination targets as reflected in practice

**DOI:** 10.1186/s12954-023-00774-1

**Published:** 2023-04-13

**Authors:** H. Stöver, A. Dichtl, D. Schäffer, M. Grabski

**Affiliations:** 1 Institute of Addiction Research, Health and Social Work, Frankfurt University of Applied Sciences, Nibelungenplatz 1, 60318 Frankfurt am Main, Germany; 2 Deutsche Aidshilfe, Berlin, Germany

**Keywords:** HIV, HCV, WHO elimination targets, Key populations, Prison

## Abstract

People who inject drugs (PWID) and prisoners are considered key populations at risk for human immunodeficiency virus (HIV) and/or Hepatitis C Virus (HCV). In 2016, the Joint United Nations Program on HIV/AIDS (UNAIDS) was implemented to eliminate HIV and AIDS by 2030 and the World Health Organization (WHO) presented the first strategy to eliminate viral hepatitis by 2030 as well. Following the objectives of the WHO and the United Nations, the German Federal Ministry of Health (BMG) presented the first integrated overall strategy for HIV and HCV in 2017. This article discusses the situation of PWID and prisoners in Germany with regard to HIV and HCV five years after the adoption of this strategy, on the basis of available data and against the background of the most recent practice in the field. In order to meet the elimination goals by 2030, Germany will have to improve the situation of PWID and prisoners substantially, mainly through the implementation of evidence-based harm reduction measures as well as the promotion of diagnosis and treatment in prisons and in freedom.

## Introduction

The vast majority of all new HIV and HCV infections in Western and Central Europe as well as in North America occur in people from particularly vulnerable groups, termed ‘key populations’ by the World Health Organisation (WHO). These key populations are defined as: men who have sex with men (MSM), sex workers, people from a migration background, transgender people, people who inject drugs (PWID) and prisoners [1, 2]. PWID and prisoners are especially affected by HIV and HCV and their vulnerabilities often overlap, which greatly maximizes the risk for individuals affected by both. For example, the majority of PWID have been incarcerated at least once [3], and use of injecting drugs in prison poses a greater risk of infection than use on the outside [4].

In 2016, the UNAIDS to eliminate HIV and AIDS by 2030 was implemented and soon after the World Health Organization presented the first strategy to eliminate viral hepatitis by 2030. Following this, in 2017, the German Federal Ministry of Health (BMG) presented the “Integrated Strategy for HIV, Hepatitis B and C and Other Sexually Transmitted Infections” (BIS 2030), for the joint elimination of both HIV and HCV by 2030. The main aims of this combined approach were to better harness similarities in disease prevention, testing and patient care as well as to pool resources.

For the elimination of HIV and AIDS the UNAIDS set three targets to be achieved by 2030: for 95% percent of those living with HIV to know their infection status, 95% percent of those who know their infection status to be on antiretroviral therapy and 95% percent of those on antiretroviral therapy to be virally suppressed. With rates of 88% of infected persons knowing their status, 96% of these on antiretroviral therapy and 96% of these virally suppressed, the targets for the general population were already partly achieved in Germany in 2019 [5]. When looking at the situation of particularly vulnerable groups, however, the overall picture is somewhat different. According to UNAIDS, vulnerable groups account for 62% of all new HIV infections worldwide and 96% of all new HIV infections in Western and Central Europe and in North America [6]. In global terms, the risk of an HIV infection is 26 times higher for MSM, 29 times higher for PWID, 30 times higher for sex workers and 13 times higher for transgender people [1, 6].

Regarding HCV, the WHO targets stipulate that 90% percent of all people infected with HCV be diagnosed and 80% percent of those in need of treatment to receive therapy by 2030. Furthermore, they specified a reduction of new HCV and Hepatitis B (HCB) infections by 90% and a reduction of HCV and HCB related deaths by 65% [1]. Unfortunately, a comprehensive assessment of the status of HCV elimination in Germany is currently not possible, because not all core and additional indicators defined by the WHO are being measured to monitor the progress. A systematic review based on a highly heterogeneous data pool estimated the overall HCV prevalence at 1.1% in countries of the European Union (EU) and the European Economic Area (EEA) [7]. With 4.3–86.3% among prisoners, 13.8–84.3% among PWID and 0–4.7% among MSM, the rates among the vulnerable groups also vary greatly, but are significantly higher compared to the general population [8]. Recent research suggests that improvements need to be made “to almost all aspects of the elimination strategy” in Germany to reach the 2030 target. Specifically, after a great initial wave of treatment response, currently most HCV positive people are not treated and this is particularly evident in key populations [9, 10].

Five years have passed since implementation of the BIS 2030 and 7 years since the approval of the latest generation of direct-acting antivirals (DAAs) to treat HCV. Today, DAA therapy takes 8–12 weeks, involving virtually no side effects and offering good chances of cure (> 95%). Since the introduction of antiretroviral therapy (ART) and its adjustments in the last decades, the quality of life of people living with HIV has improved significantly and opportunistic infections have become more rare. Despite these favourable basic conditions, the goal of eliminating HCV and HIV in Germany still seems far removed, especially in key populations. In the following sections, the present situation of PWID and prisoners as groups particularly affected by HIV and HCV is evaluated on the basis of the currently available data. In addition, target group-specific developments in the field are presented as examples and taken as the basis for a careful interim status report and an outlook on the road to 2030. The focus on PWID and prisoners will represent a fundamental requirement for meeting the target of eliminating HIV and HCV by 2030.

### Eight years to go until 2030: current situation among PWID

After years of decline, the percentage of PWID among all new HIV infections reported annually in Germany has increased continuously from 6% in 2012 to 13.8% in 2019. In 2019, about 360 people became infected with HIV through injecting drug. Altogether 10.8% of all people living with HIV in Germany have become infected through injecting drug use [5].

Injecting drug use was indicated in 2019 as the most probable mode of transmission of HCV infection (64%) where information on the mode of transmission was available. Thus, PWID are by far the group most vulnerable to HCV [11]. In 2.9% of the cases, the infection most likely occurred in prison [11]. Overall, it should be considered that the entirety of the cases includes a large number of chronic HCV cases, since the majority of new infections are asymptomatic and the reference definition comprises all HCV infections that are detected in the laboratory for the first time. 79% of all cases in 2019 where information on the disease stage was available were already chronic cases.

The DRUCK study collected supraregional data on HIV and HCV from PWID in eight German cities between 2011 and 2014, some revealing very high prevalence rates: The prevalence of HIV ranged between 0 and 9%; a total of 80% of those tested positive were aware of their diagnosis and 55% were receiving antiretroviral therapy at that time [12]. The antibody prevalence of HCV ranged between 42 and 75%, with an active infection (RNA-positive) being present in 23–54% of all study sites. 85% of people infected with HCV in whom a treatment was indicated were aware of the infection and 19% reported that they had undergone a successful therapy [3].

Another study that collected data in 2010/2011 established a prevalence of HIV positive infections of 4.8% among drug users in the community and 6.2% among those in the clinical environment based on self-reports. With 58.7% and 58.3% the prevalence of active HCV infections ranged at a similarly high level in the community and the clinical environment, respectively [13].

In addition to the data reported to the RKI (Robert Koch Institute) and the results of the DRUCK study, annually collected data from the Hamburg outpatient addiction treatment monitoring system (BADO) are also available [14]. According to these data among drug users the prevalence of active HCV infection was 39% and of positive HIV infections 4%. In this case drug use was defined as seeking help at the facility for the use of at least one substance. The consumption room documentation from Frankfurt am Main, which collects self-reported data, reported that the prevalence of positive HIV infections among consumption room users was 2.7% and the prevalence of active HCV infection was 24%. In Hamburg, the HIV infection rate slightly declined over the last 10 years; the HCV infection rate was stable until 2016 and has then declined until 2018. In Frankfurt, the reported HIV rate has been stable with slight fluctuations since 2012 and the HCV rate has been decreasing since 2012.

The pilot project “HIV? Hepatitis? I CHECK that!” was conducted by the BZgA (Federal Centre for Health Education) in collaboration with the RKI and the DAH (German AIDS Service Organisation) and included both counselling and HIV/HCV testing for drug users in low-threshold facilities in six German cities. Here 26.8% of the tested participants had an active HCV infection and a further 9.4% a positive antibody result without further information on whether the infection was still active or not. The prevalence of persons confirmed positive for HIV over the course of the project was 0.4% (2 out of 430 tested persons) [15].

## Eight years to go until 2030: current situation among prisoners

Since the mid-1980s, prisons have been increasingly confronted with viral and bacterial infectious diseases such as HIV/AIDS, hepatitis (A, B and C) and/or tuberculosis. Compared to the general population, current and former PWID and people infected with HCV/HIV are clearly overrepresented in German prisons [16] and in prisons globally [17]. The available data on HIV and HCV among prisoners is even more limited than in other key populations. The first Germany-wide survey on the problem of substance addiction within the correctional system from 2018 demonstrated that 44 percent of the 41,896 prisoners covered had a substance use disorder (dependence and abuse according to the criteria of the WHO ICD-10) at the time of starting their prison sentence. 27% were affected by dependence and 17% by harmful use of psychotropic substances [18]. A substantial part of the prisoners also continued to use drugs in prison [19]. Studies have demonstrated that 30% of former prisoners injected drugs while in prison, whereas 11% started injecting drugs during imprisonment [3, 4]. Moreover, imprisonment represents a risk factor for starting drug use. Infectious diseases are among the major health disorders in prisons. Compared to the general population, prisoners are 48–69 times more likely to be infected with HCV and 7–12 times more likely to be infected with HIV [20]. In a cross-sectional study 17.6% of the examined prisoners had an HCV infection and 0.8% an HIV infection [21]. About half of the prisoners who had ever injected drugs were HCV-positive and 1.6% were HIV-positive. A study from 2009 by Schulte et al. revealed similar results; the percentage of PWID among all prisoners was 21.9%. The overall HCV prevalence was 14.3%, of which 5.5% received treatment last year. The HIV prevalence was 1.2%, of which 86.5% were on antiretroviral therapy [22].

As part of the DRUCK study the vast majority (81%) of participants indicated that they had been imprisoned at least once in life, 32% of them within the last year. The median overall term of imprisonment was three years and six months [23]. This is in line with other German studies asking about experiences of incarceration [24]. Nearly one-third of the respondents indicated that they had injected drugs while in prison. 54 participants (0.6–6% of the participants from the various study cities) reported that they had started to inject drugs in prison. In addition, an association between HCV infections and incarceration was shown: the HCV prevalence increased with increasing duration and frequency of imprisonment. A recent publication has confirmed the association between HCV/HIV infections and imprisonment for PWID in 17 European countries [25].

For many years, there has been no supra-regional study on HIV/HCV in prisons in Germany. And a systematic nationwide screening does not exist either. However, some Länder and prisons with different testing strategies regularly collect data on infectious diseases among prisoners. In 2020, for example, a total of 13,403 RNA blood tests were carried out in Bavarian prisons (12,389 in men and 1014 in women), of which 77 were positive for HIV (72 in men and 5 in women) and 1081 were positive for HCV (1006 in men and 75 in women) [26]. In the 2019 reporting year, 21 HIV positive and 273 active HCV cases were detected among prisoners voluntarily tested for HIV and HCV in prisons in the state of Baden-Württemberg [27].

Regarding the treatment prevalence in German prisons, Müller et al. [28] have come to the conclusion that HIV treatment is the only one of four investigated treatments (HIV, HCV, tuberculosis and osteoarthritis [OST]) that is offered to an extent appropriate to the estimated number of infected prisoners. By contrast, the prevalence of HCV treatments was found to be too low, as was the number of substitution therapies in some Länder [29, 30]. Great differences in the provision of treatments were observed between the Länder, suggesting inconsistent HCV policies and practices in German prisons, despite extramural treatment guidelines being in place [31]. Consequently, medical care under the principle of equivalence (equal quality of medical care within and outside of prison) is not implemented universally in all Länder (see also [16, 32, 33]).

### Current approaches to eliminating HIV and HCV among drug users and prisoners in Germany

In retrospective, the DRUCK study can be seen as ground-breaking in terms of HIV and HCV elimination in PWID in Germany. This study collected data on risks of infection, knowledge and behaviours of injecting drug users by means of a sero-behavioural survey. After piloting in 2011 in Berlin and Essen, the study was carried out in six further cities from 2012 to 2015. The study’s primary goal was to establish the prevalence of HBV, HCV and HIV as well as co-infections among PWID in Germany and the secondary aim was to determine the influence factors for HBV, HCV and HIV among PWID in Germany as well as possible knowledge gaps among PWID with regard to the transmission and prevention of these infections. The most important recommendations derived from the study were: (1) demand-oriented distribution of drug use equipment, (2) targeted brief counselling on HCV/HIV transmission routes, (3) establishment of HIV/HCV counselling and testing services in low-threshold facilities, (4) targeted information for substituted patients and doctors on immunisation, HCV/HIV testing and treatment, (5) regular training for the qualification of (non-medical) staff in drug counselling centres as consultants, and (6) confidential and voluntary HCV and HIV testing in prisons. Following the DRUCK study and in an effort to implement the objectives of the national “BIS 2030” strategy, these recommendations were taken up and extensively addressed by the Federal Government and collaborating NGOs, as demonstrated by the examples below.

This includes the development of recommendations for the distribution of drug use equipment as part of an action plan of Deutsche AIDS-Hilfe (DAH) [34]. Based on the results of the DRUCK study, the SMOKE IT 2 study was additionally conducted [35]. The study was aimed at raising awareness of inhaling drug use as a harm reduction and infection prevention measure in drug counselling centres and AIDS service organisations and supporting a temporary or permanent change in the form of application (from injecting to inhaling) among heroin users. Multimedia campaigns (videos, print media, prevention tools) designed to change the form of consumption were carried out during and after the study [36]. In addition, DAH focused its media work for drug users on HIV and HCV prevention, and messages designed to reduce the lack of knowledge regarding the risks of sharing drug use equipment other than syringes and needles, as identified by the DRUCK study. For the first time, these media were also produced in Farsi, Arabic and Russian [37]. The BZgA supported these measures over a period of 3 years by providing facilities with the opportunity to order free smoking foils from DAH. These measures enabled facilities throughout Germany to add foils to their range of drug use equipment. The possibility of free ordering accompanied by brief interventions and media campaigns contributed to raising the profile of inhaling drug use. Simultaneously, a significant decline in injecting use in favour of inhaling and nasal forms of consumption has been observed in recent years [38]. To what extent the studies have contributed to this trend cannot be assessed at present.

The establishment of HIV and HCV counselling and testing services in low-threshold facilities was one of the core objectives of the DRUCK study. With the pilot project entitled “HIV? Hepatitis? I CHECK that!” conducted by the BZgA, the RKI and DAH, counselling and testing services in AIDS service organisations and drug counselling centres were integrated with low-threshold services to enable active drug users to make use of free and anonymous HIV and HCV tests [39]. At a total of six locations in 4 Länder, drug users were offered weekly counselling on HIV and hepatitis, high-risk situations and possibilities of prevention as well as free and anonymous rapid and laboratory tests for 20 months. The results made clear that counselling and testing outside medical practices is necessary, is well received by drug users and can be offered in high quality. Despite the positive results of the accompanying scientific research, it has so far not been possible to convince the Länder to continue and expand this project. Only in Hamburg further funding was received by the city parliament so that it could continue beyond the pilot phase.

Simultaneously to studies, pilot interventions and media, the above-mentioned recommendations were also implemented in further education. For example, a consultant training course to prepare staff members of AIDS service organisations and drug counselling centres for the implementation of counselling and testing projects was integrated into the seminar programme of DAH. Furthermore, a training and practical manual was developed by the umbrella organisations DAH and AIDS relief from the state of North-Rhine Westfalia using the expertise gained from the fieldwork. This can contribute to raising the levels of knowledge on HIV, hepatitis and STIs among staff members of low-threshold drug support facilities and providing guidance on the methodological, financial, personnel and spatial implementation of a counselling and testing project. The available 500 copies of the print version were out of print within one year, demonstrating that AIDS service organisations and drug counselling centres have a high interest in implementing their own testing projects.

In March 2020 the requirement for antibody testing to be performed by medical professionals was eliminated with the goal of enabling AIDS service organisations and drug counselling centres to carry out testing projects without medical staff [40]. The DAH saw an abrupt rise in the number of facilities interested in subject-specific further training. In 2020 alone, 30 on-site training sessions were offered, but were only implemented sporadically due to the Covid-19 pandemic. However, the high level of interest demonstrates that this change in law could increase the number of low-threshold facilities offering HIV and HCV rapid testing accompanied by counselling services as well as referral to specialists.

The lack of testing services in the context of substitution therapy was once again addressed by an information campaign of the BZgA with the involvement of professional associations and self-help organisations. A fact sheet was sent to all doctors specialising in addiction treatment and to more than 8000 general practitioners. Additionally, the campaign comprised a brief information sheet for patients as well as waiting room posters to make the patients aware of the option of free testing. However, an assessment as to whether the awareness of HIV and hepatitis diagnoses has been raised in addiction medicine as a result cannot be made yet.

Recognised prevention activities have so far been only sporadically implemented in German prisons. This is also established in the “BIS 2030” strategy of the Federal Government: “Recognised prevention activities, including medication-assisted treatment for opioid users, or the provision of condoms and lubricant gel, can minimise the risk of HIV, hepatitis B and C transmission. These have not yet been equally and universally implemented or made available.” [41].

As described above, opioid substitution therapy for opioid-dependent prisoners is not offered universally, although its positive effects with regard to the prevention of infectious diseases, the reduction of opioid use [42], and the reduction of mortality after release from prison have been verified [43, 44].

In view of the high prevalence of HIV,AIDS,HCV and HBV, the call for the introduction of needle and syringe programmes in prisons has repeatedly been made by professional associations and experts from a professional and legal point of view (see also [19, 45–47]). However, it is refused by most ministries of justice in Germany predominantly for reasons of penal policy. In 1996, pilot projects started in some German prisons in Lower Saxony, Hamburg and Berlin. Since 2001, however, six of the seven projects have been discontinued for political reasons [48, 49]. The Lichtenberg women’s prison in Berlin is now the only one of 181 prisons to offer a needle and syringe programme, even though practical experience and scientific evaluations consistently prove the success of such measures [46, 50–53].

HIV and HCV testing as part of the initial medical examination in prisons and the initiation of medical treatment are not universally available in German prisons [22]. As a consequence undiagnosed HIV and HCV infections cannot be treated. The number of HCV treatments in prisons is extremely low to begin with [27]. In this context, another recommendation of the DRUCK study was implemented in 2016 by DAH initiating a pilot project for HIV and HCV counselling and testing in the Tonna correctional facility with the AIDS service organisations in the state of Thuringia. The existing concept could be used as a blueprint for implementation in other prisons in other Länder.

A large number of sexual contacts, either consensual or forced, are documented in prisons. They mostly take place unprotected [52]. However, the anonymous and discreet provision of condoms and lubricants called for by experts is only implemented in prisons to a very limited extent, with great differences in the distribution models: From sale to distribution by doctors and care staff upon request only, a wide range of sometimes very high-threshold approaches can be observed in German prisons [54].

### Leave no one behind: a long way to go until 2030

Since the introduction of the numerous new DAAs against HCV in 2014, the basic conditions for HCV elimination in Germany have been very favourable. And since the introduction of antiretroviral therapy (ART) and its adjustments, the lives of people with HIV have also been improved significantly and opportunistic infections have become less common. Despite the positive developments in treatment and the fact that the WHO elimination targets for HIV have almost been accomplished in the general population, they are still far from being achieved for HCV in general as well as for HCV and HIV among the key populations of PWID and prisoners. Assuming that roughly 9900 HCV treatments are performed annually, Germany is currently just barely keeping up with those countries that are on track to meet the WHO HCV targets by 2030 [55].

Offering prevention and care services tailored to the main target groups of PWID and prisoners as two of the key populations could potentially contribute to meeting the targets on a national scale. Since the adoption of the strategy, numerous independent agencies and associations have promoted mainly selective improvements for PWID in freedom. In some cases this was done in collaboration with government institutions, but in many cases independently in pilot projects and examples of good practice. Despite the wide range of measures developed in recent years and the well-structured interdisciplinary implementation of the recommendations of the DRUCK study, comprehensive and substantial progress has not been made.

There is some hope regarding the identified lack of data on PWID and prisoners as the new DRUCK 2.0 study by the RKI aims to implement an HIV and HCV monitoring system for drug users and might be able to fill the current gap. At the European level, the Correlation-European Harm Reduction Network (C-EHRN) is already making an important contribution by means of a monitoring tool that reflects the insights of civil society and takes a look at political measures concerning HCV and PWID as well as the HCV continuum of care for PWID [56]. It concluded that the elimination targets for the key population of PWID can only be achieved by involving civil society drug support and self-help organisations in planning a continuum of care of HCV testing and treatment. Despite the efforts undertaken by the Federal Government and professional associations, it has so far not been possible in Germany to demonstrate the significance of HIV and HCV (rapid) testing services in low-threshold drug support facilities to the. The challenges posed by the high workload and the low financial resources of drug support facilities, and the strict separation of medicine and social work can only be overcome if the corresponding financial resources are made available for the establishment of a whole new range of services. This is incumbent upon the municipalities and the Länder.^1^ Alongside the expansion of testing and treatment services for PWID, the implementation of indirect and direct infection prevention measures must also be expedited. To reduce the incidence and prevalence of HCV among PWID, having access to low-threshold substitution therapy is indispensable, given that it has proved effective in preventing HCV infections and can reduce the HCV incidence by more than 70% in combination with high-coverage needle and syringe programmes [57].

Since the adoption of the “BIS 2030” strategy, there has also been no apparent improvement in the situation of people in prison. According to the principle of equivalence [58, 59] prisons have the duty to adopt the public health goals and to provide people in prison with sufficient testing, prevention and treatment options. Regarding the available data on the prevalence of infectious diseases among people in prison, the ministries of justice at Länder level have so far shown little involvement and determination in tackling this issue. The objectives of the “BIS 2030” strategy were identified and specified only in isolated cases, such as in the final report of the expert committee on the further development and improvement of medical care in the state of Baden-Württemberg prisons [27]. The focus in prisons should also be placed on expanding the testing and treatment services and implementing indirect and direct infection prevention measures. A substantial expansion of medication-assisted treatment in prisons and the distribution of drug use equipment to all drug users by ensuring low-threshold permanent access to syringe vending machines are required to prevent infections in prisons and counter high-risk forms of consumption. Post-exposure prophylaxis (PEP) after needlestick injuries and/or in emergency cases of risk exposure through multiple use of contaminated injection equipment should also be made available as a standard intervention in prison medical services. PEP can still prevent an HIV infection with a high probability. Free, low-threshold, confidential and anonymous access to condoms and water-soluble lubricants should also be ensured in all prisons.

To achieve the 2030 elimination targets, Germany will have to significantly step up its efforts and focus on currently underserved key populations, as in this case PWID and people in prison, especially against the background of the delayed implications of the COVID-19 pandemic. The foundation for a common objective and coordinated approach between the Federal Government, the Länder, municipalities and drug service organisations must be the implementation of evidence-based harm reduction measures and the promotion of diagnosis and treatment within and outside prisons. Furthermore, the coordination and cooperation between the health and the justice stakeholders on all levels is imminent.

If PWID and people in prison are not included on the road to 2030, Germany will most likely fail to meet the elimination targets.

## Data Availability

We used publicly available data and had no problems in accessing these data.

## Footnotes

1 A network of so-called checkpoints has already been established for the target group of MSM. In about 50 cities, AIDS service organisations, prevention projects and other facilities offer HIV, STI and HCV counselling and testing services with great success.
